# Dual-Tasking in the Near-Hand Space: Effects of Stimulus-Hand Proximity on Between-Task Shifts in the Psychological Refractory Period Paradigm

**DOI:** 10.3389/fpsyg.2018.01942

**Published:** 2018-11-06

**Authors:** Thomas J. Hosang, Rico Fischer, Jennifer Pomp, Roman Liepelt

**Affiliations:** ^1^Department of Performance Psychology, Institute of Psychology, German Sport University Cologne, Cologne, Germany; ^2^Experimental Psychology Unit, Department of Psychology, Helmut Schmidt University, Hamburg, Germany; ^3^Department of Psychology, University of Greifswald, Greifswald, Germany; ^4^Department of Psychology, Dresden University of Technology, Dresden, Germany; ^5^Institute for Psychology, University of Münster, Münster, Germany

**Keywords:** dual task, cognitive control, psychological refractory period (PRP), multitasking, near-hand space, embodied cognition, attention, peripersonal space

## Abstract

Two decades of research indicate that visual processing is typically enhanced for items that are in the space near the hands (near-hand space). Enhanced attention and cognitive control have been thought to be responsible for the observed effects, amongst others. As accumulating experimental evidence and recent theories of dual-tasking suggest an involvement of cognitive control and attentional processes during dual tasking, dual-task performance may be modulated in the near-hand space. Therefore, we performed a series of three experiments that aimed to test if the near-hand space affects the shift between task-component processing in two visual-manual tasks. We applied a Psychological Refractory Period Paradigm (PRP) with varying stimulus-onset asynchrony (SOA) and manipulated stimulus-hand proximity by placing hands either on the side of a computer screen (near-hand condition) or on the lap (far-hand condition). In Experiment 1, Task 1 was a number categorization task (odd vs. even) and Task 2 was a letter categorization task (vowel vs. consonant). Stimulus presentation was spatially segregated with Stimulus 1 presented on the right side of the screen, appearing first and then Stimulus 2, presented on the left side of the screen, appearing second. In Experiment 2, we replaced Task 2 with a color categorization task (orange vs. blue). In Experiment 3, Stimulus 1 and Stimulus 2 were centrally presented as a single bivalent stimulus. The classic PRP effect was shown in all three experiments, with Task 2 performance declining at short SOA while Task 1 performance being relatively unaffected by task-overlap. In none of the three experiments did stimulus-hand proximity affect the size of the PRP effect. Our results indicate that the switching operation between two tasks in the PRP paradigm is neither optimized nor disturbed by being processed in near-hand space.

## Introduction

The human visual system evolved to not only perceive the world, but also to enable physical interaction with the environment (Goodale, 2011). More than 20 years of research support this reasoning, showing altered visual processes close to one of the main human effectors, the hands. Typically, performance is assessed using different stimulus-hand proximities, comparing a condition in which stimuli are presented close to the hands (near-hand condition) and a condition in which stimuli are presented further away from the hands (far-hand condition). Earliest accounts of altered visual processing in near-hand space was provided by Hari and Jousmaki (1996), showing faster reaction times (RTs) when visual stimuli were presented near the hands. Their results indicated prioritized visual processing of stimuli in the near-hand space (near-hand effect). A number of neuropsychological studies subsequently provided supporting findings for this effect, reporting improved visual processing in the near-hand space in patients with extinction (di Pellegrino et al., 1997; di Pellegrino and Frassinetti, 2000) and hemianopsia (Schendel and Robertson, 2004).

Since these findings were obtained, considerable effort has been put into exploring behavioral performance in healthy individuals during visual cognition tasks in the near-hand space (for reviews see Tseng et al., 2012; Brockmole et al., 2013; Abrams et al., 2015; Goodhew et al., 2015; Thomas and Sunny, 2017). Study findings have shown that, for example, task processing in near-hand space includes increased visual working memory performance (Tseng and Bridgeman, 2011), emphasized magnocellular information processing (Gozli et al., 2012; Goodhew et al., 2014), enhanced cognitive control (Wang et al., 2014; Weidler and Abrams, 2014; Liepelt and Fischer, 2016), and enhanced attention (Reed et al., 2006; Abrams et al., 2008). Moreover, visual processing in the near-hand space can be biased, not only by the mere presence of the hands, but also by the specific hand-posture (Thomas, 2015), plasticity (Makin et al., 2010; Reed et al., 2017; Thomas, 2017), and task-demands (Goodhew and Clarke, 2016; Liepelt and Fischer, 2016). In summary, the available literature indicates that stimuli and tasks are processed differently in the near-hand space. These effects can be traced back to diverse alterations that range from changes in early perceptual processing to changes in cognitive control (Tseng et al., 2012; Brockmole et al., 2013; Abrams et al., 2015; Goodhew et al., 2015; Thomas and Sunny, 2017).

It is important to note that almost all of the evidence for the near-hand effect comes from single-task experiments, in which only one stimulus is attended to and only one task is processed. Cognitive control and attentional processes, among others, were held responsible for the observed effects. There is accumulating experimental evidence (Liepelt et al., 2011; Fischer and Hommel, 2012) and theoretical rationale (Meyer and Kieras, 1997; Logan and Gordon, 2001) that suggests the involvement of cognitive control processes during the scheduling and coordination of two simultaneous tasks (dual tasking). If near hand space alters cognitive control and attention (Abrams et al., 2008; Wang et al., 2014; Weidler and Abrams, 2014; Liepelt and Fischer, 2016) and between-task shifts during dual tasking involve cognitive control and attention (Meyer and Kieras, 1997; Logan and Gordon, 2001; Luria and Meiran, 2003; Koch et al., 2018), one should predict a modulation of dual-tasking performance in the near-hand space as compared to far-hand space. Also, societal and technological advances have increased the demands on multimedia multitasking and the complexity of human-technological interactions. The common use of hand-held devices, for example, shifts the visual-manual interaction into a single visuo-spatial region. To date, it remains unclear how the near-hand space affects one’s processing of multiple stimuli in the visual display that are assigned to different tasks. The aim of the present study is to investigate the impact of stimulus-hand proximity in a dual-task situation in which the stimulus (Stimulus 1) of Task 1 is presented on the right and requires responses with the right hand and the stimulus (Stimulus 2) of Task 2 is presented on the left and requires responses with the left hand. We use the psychological refractory period (PRP) paradigm to test the efficiency of the shifting process between Task 1 and Task 2 processing under dual-task conditions. The PRP paradigm allows for an exact assessment of Task 1–Task 2 shifts due to the precise experimental manipulation of the temporal overlap of two tasks. The better Task 2 performance at short SOAs (i.e., indexed by the size of the PRP effect), the more efficient the engagement of Task 2 processing. The PRP paradigm thus represents a perfectly suitable approach to precisely measure the shifting operation in dual-task contexts.

It has previously been indicated that, in single-task studies, the benefit of increased in-depth visual processing of an attended stimulus comes at the cost of delayed disengagement from this stimulus (e.g., Abrams et al., 2008). For example, the effects of inhibition of return (i.e., costs of re-allocating attention to previously engaged locations) have been shown to be decreased in near-hand space, a finding that was interpreted as slower disengagement from the originally attended location of stimulus processing. This interpretation has been further substantiated by the findings of Abrams et al. (2008) showing an increased attentional blink effect in near-hand space (Abrams et al., 2008). The attentional blink characterizes the inability to detect a second target presented in rapid succession to a first one (Raymond et al., 1992; Shapiro et al., 1997). In particular, participants were required to report the parity of a digit (Stimulus 1) and then the identity of a letter (Stimulus 2) that was presented at various intervals following Stimulus 1. While the typical pattern of the attentional blink was found in the far-hand condition, this inability to detect Stimulus 2 within short succession of Stimulus 1 was much more pronounced when participants’ hands were close to the stimuli. Taken together, these findings suggest that increased in-depth visual processing of an attended stimulus in near-hand space might result in costs when switching the processing of one stimulus to another stimulus.

The findings from a sequential dual-task study (i.e., task switching) by Weidler and Abrams (2014) are, however, quite the opposite. The authors, suspecting an increased engagement of cognitive control processes in near-hand compared to far-hand conditions, tested a task-switching paradigm. Participants were presented with bivalent stimuli (i.e., colored geometrical figures) while a cue indicated which task had to be performed (i.e., color or shape discrimination). The important factor was the repetition or alternation of task type, as there are typically larger performance costs when tasks alternate rather than repeat. Such task switching costs are thought to be a marker for flexible updating and reconfiguration of task sets (Monsell, 2003; Kiesel et al., 2010; Vandierendonck et al., 2010; Koch et al., 2018). Importantly, Weidler and Abrams (2014) found reduced switching costs in near-hand compared to far-hand conditions. These findings were interpreted as evidence for an increased level of cognitive control involvement during near-hand conditions. Although this is in line with other reports of increased cognitive control in near-hand space (e.g., Davoli et al., 2010; Liepelt and Fischer, 2016), the mechanisms by which stimulus-hand proximity might reduce switching costs have not yet been identified. Current explanations range from an increased maintenance of task instructions to the activation of the correct S-R translation rule (Weidler and Abrams, 2014) due to enhanced cue processing. In any case, these findings show that shifts between two different task sets seem to be less costly when stimuli are presented in near-hand space.

Overall, the existing literature offers only inconclusive assumptions with regard to the question how the processing of multiple stimuli might be affected when stimuli are presented in near-hand space and how this differs from far-hand conditions. In dual tasks, processing of the stimulus in Task 1 is accompanied by additionally processing the stimulus in Task 2. While early perceptual processes might occur at the same time, at some point processing must shift from Task 1 to Task 2 (see below for more details). If each stimulus is spatially presented to a separate response hand (e.g., Stimulus 1 near the right hand and Stimulus 2 near the left hand), it remains unclear how hand proximity affects this processing shift between tasks.

Evidence from visual attention studies (e.g., Abrams et al., 2008) suggests that near-hand beneficial processing of Stimulus 1 results in delayed disengagement. Hand-nearness facilitates attentional processing of the respective stimulus (e.g., Stimulus 1). This however, might induce cost when shifting processing from Stimulus 1 to Stimulus 2 is required in a dual task. Evidence from task switching studies, however, indicates the opposite. Reduced task switching costs in near-hand space suggest beneficial switching between different task sets (Weidler and Abrams, 2014). Here, the attentional benefit of processing stimuli in near hand space might extend to both, Stimulus 1 and Stimulus 2, easing the shifts between the two stimuli. Thus, by investigating dual-task performance in different stimulus-hand proximity conditions, we learn whether and to which extent the attentional consequences of hand nearness affect the processing shift between two tasks.

In the present study, we apply a PRP dual-task paradigm that allows the investigation of simultaneous task component processing. In particular, two RT tasks are presented with varying temporal intervals [stimulus onset asynchrony (SOA)]. Participants are instructed to respond with their right hand to an initial visual stimulus (Stimulus 1) presented on the right and then to respond with their left hand to a second stimulus (Stimulus 2) presented on the left. Whereas Task 1 processing is mostly unaffected by SOA, RTs for Task 2 typically increase with decreasing SOA between both tasks. Impaired Task 2 performance at short compared to long SOA is known as the PRP effect (Welford, 1952; Pashler, 1994). The PRP effect is commonly attributed to a capacity limitation (e.g., processing bottleneck) and it is assumed that a central cognitive stage in Task 1 has to be completed before processing of that stage of Task 2 can proceed. Although the existence of a processing bottleneck is widely accepted, there is still a debate over its exact nature (i.e., whether it is structural, strategic, or functional) (Pashler, 1994; Meyer and Kieras, 1997; Logan and Gordon, 2001; Tombu and Jolicœur, 2003; Fischer and Plessow, 2015; Broeker et al., 2017). As with task switching, the involvement of cognitive control processes in scheduling and coordinating the simultaneous performance of two tasks has been advocated by many authors (Meyer and Kieras, 1997; Logan and Gordon, 2001; Luria and Meiran, 2003; Liepelt et al., 2011; Koch et al., 2018). Even though task priority is typically given to the first task (Task 1), at a given point in time task processing has to shift to Task 2, which can occur passively (Pashler, 1994) or can be realized by cognitive control parameters optimizing task (dis)engagement (Meyer and Kieras, 1997; Logan and Gordon, 2001). Currently, it is not clear whether near-hand space affects this Task 1–Task 2 processing shift in dual tasking. This is surprising given that the processing of multiple stimuli and tasks is an increasingly prevalent aspect of daily human-technology interaction. Investigating the effects of stimulus-hand proximity on PRP performance holds the potential to get further insights into how stimulus-hand proximity and corresponding changes in cognitive control affects switching operations during the PRP paradigm (Meyer and Kieras, 1997; Logan and Gordon, 2001).

If the near-hand space results in delayed disengagement from processing the prioritized Stimulus 1 (Abrams et al., 2008), shifts from Task 1 to Task 2 processing should be prolonged, resulting in an increased PRP effect for near-hand compared to far-hand conditions. Alternatively, if the near-hand space facilitates switching between two tasks sets (Weidler and Abrams, 2014), shifts from Task 1 to Task 2 processing should benefit from near-hand conditions. This should result in a reduced PRP effect when hands are located near the stimuli.

## Experiment 1

In Experiment 1, we used a dual-task paradigm adapted from the PRP literature (Fischer et al., 2007). The paradigm was chosen to specifically test the effect of the near-hand space on Task 1–Task 2 switching by means of the PRP effect. Task 1 was a number categorization task wherein numbers had to be categorized into either odd or even. For Task 2, participants had to perform a letter categorization task wherein letters had to be categorized as either a vowel or a consonant. Stimulus 1 was first presented on the right side of the screen and required responses with the right hand. Stimulus 2 appeared on the left side and required responses with the left hand. For the near-hand condition response buttons were placed on the monitor. For the far-hand condition response buttons were placed on the lap.

### Methods

#### Participants

Thirty-six participants from the Dresden University of Technology (28 female; *M*_age_ = 25.1 years, *SD* = 5.6) were tested. Participants received either course credits or monetary reward for their participation. All participants had normal or corrected-to-normal vision. All but three of the participants claimed to be right-handed. Written informed consent was provided by all participants prior to their participation in the experiment. All experiments were conducted in accordance with the ethical standards of both the 1964 Declaration of Helsinki and the German Psychological Association.

#### Design

A 2 (stimulus-hand proximity: near vs. far) × 4 (SOA: 40, 130, 300, and 900 ms) within-subjects repeated measures design was applied.

#### Stimuli and Apparatus

**FIGURE 1:**
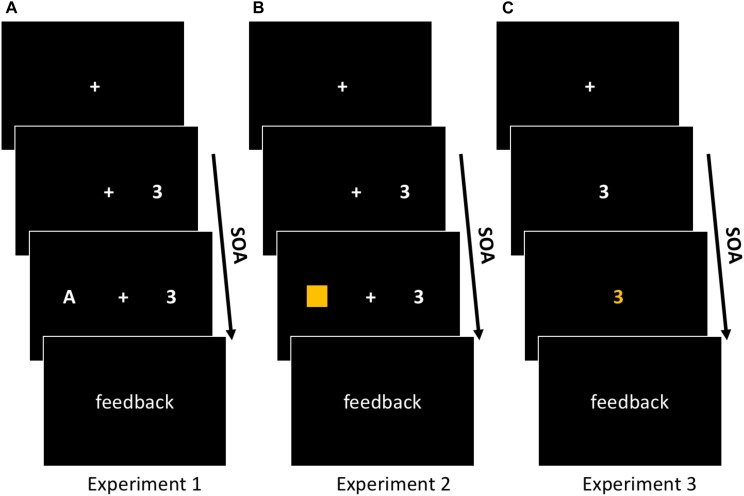
Schematic illustration of the three PRP experiments. In all three experiments Task 1 and Task 2 were presented with varying stimulus-onset asynchrony (SOA). **(A)** In Experiment 1, participants had to categorize digits (2, 3, 7, 8) into odd or even (Task 1) and letters (A, K, M, U) as either vowels or consonants (Task 2). **(B)** In Experiment 2, Task 1 was the same as in Experiment 1. Task 2 was a color-categorization task, where the color of a rectangle had to be categorized into either orange or blue. **(C)** In Experiment 3, Task 1 and Task 2 were the same as in Experiment 2, but they were presented as a single bivalent stimulus. The number stimulus relevant for Task 1 changed its color initiating the color categorization for Task 2. In all of the three experiments trials began with a fixation and ended with the provision of feedback in the form of the German words *richtig* (correct), *falsch* (incorrect), or *zu langsam* (too slow).

The digits 2, 3, 7, and 8 served as Stimulus 1 for Task 1. Stimulus 1 were presented on the right side (+5.3 cm from screen center) of a 17-inch TFT-monitor (1280 × 1024 pixel resolution). For Task 2 the letters A, K, M, and U were used as Stimulus 2. Stimulus 2 were presented on the left side of the computer screen (-5.3 cm from screen center), see Figure 1A. All stimuli were presented on a black background in Arial font (white). The viewing distance was set to approximately 45 cm, while the total presentation field extended to a visual angle of 14.2° horizontally and 1.3° vertically. The visual angle of all the the stimuli extended to 0.76° horizontally and 1.27° vertically.

**FIGURE 2:**
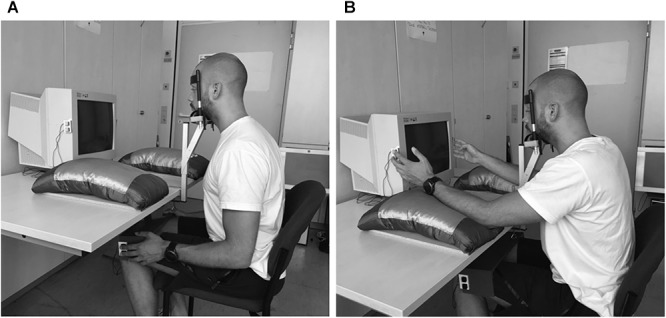
Experimental setup during the far-hand condition **(A)** and the near-hand condition **(B).**

Two manual response buttons were assigned to each hand (see Figure 2). Elbow angle, as well as the distance and spatial orientation of the response buttons was held constant between the near-hand condition and the far-hand condition. Participants responded with the index (odd numbers) and middle finger (even numbers) of their right hand to Stimulus 1 and with the index (vowel letters) and middle finger (consonant letters) of their left hand to Stimulus 2. The index fingers activated the upper buttons, while the middle fingers activated the lower buttons. For the near-hand condition the buttons were vertically arranged on the right and left sides of the computer monitor. Button placement matched the height of stimulus presentation. For the far-hand condition, the response buttons were analogously positioned on the left and right sides of a wooden board that rested on the participants’ knees. The distance between left and right response buttons of the far-hand condition (board) was matched to that of the near-hand condition (monitor).

Presentation of the stimuli and data recording was carried out using the Software Presentation (Version 16.5; Neurobehavioral Systems, Inc., Albany, CA, United States) on a Windows PC (Win7, Intel Core i5-6500 [2.6 GHz, 6MB]).

#### Procedure

A fixation started each trial (500 ms; central white cross). Stimulus 1 presentation (right side of the screen) was followed by Stimulus 2 presentation (left side of the screen) with varying temporal interval (SOA, 40, 130, 300, and 900 ms). Stimulus 1 and Stimulus 2 remained on the screen for 1000 ms. Button press in response to Stimulus 1, and Stimulus 2 initiated the ending of the trial. No response or a single button press response resulted in the abortion of the trial after a maximum of 2500 ms after Stimulus 2 offset. Feedback was given in the form of the German words *richtig* (correct), *falsch* (incorrect), or *zu langsam* (too slow) for the duration of 500 ms. Subsequent trials started after a random and variable (100–1000 ms in steps of 100 ms) response-fixation interval.

Subjects were instructed to perform Task 1 and Task 2 as fast and as accurate as possible while processing priority was instructed on Task 1. They were further instructed to not delay Task 1 response to avoid response grouping. Task 1 was a number categorization task (odd vs. even) and Task 2 was a letter categorization task (vowel vs. consonant).

The experiment had a near and far-hand condition, each comprising 3 blocks. One block contained 64 trials (16 trials per SOA). Thus, 192 trials were performed per stimulus-hand proximity condition, which equals 384 trials in total. Both conditions started with a familiarization phase (16 practice trials). During this phase the instructor was present, answered questions, and ensured that the hand position was correct. A brief break was given after each block.

### Results

**FIGURE 3:**
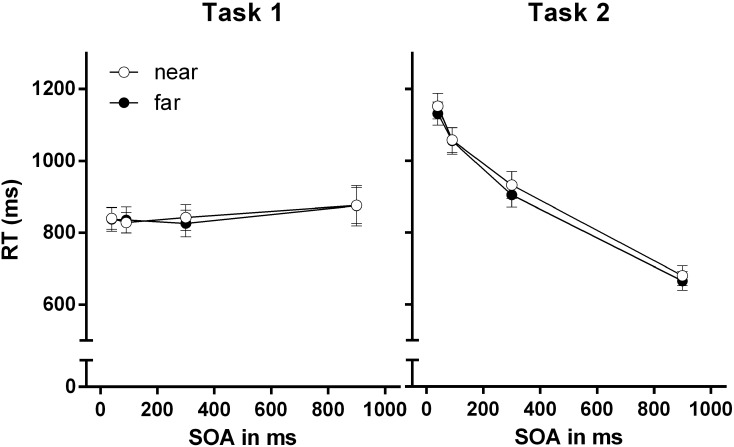
Reaction times (RTs) for Task 1 and Task 2 for the near-hand and far-hand condition in Experiment 1. Error bars represent standard errors of the mean.

A repeated measures ANOVA was conducted on RTs and percent error (PE) in both tasks and included the within-subject factors stimulus-hand proximity and SOA. For RT analyses (Task 1 RTs and Task 2 RTs) error trials in either task (9.2%), and trials that were below 150 ms or above 3000 ms (<0.1%) were excluded prior to analysis. Double-errors (Task 1 and Task 2 errors) were excluded prior to Task 2 error analyses (<1.1%). Greenhouse–Geisser correction was applied in case of violation of sphericity. RTs and PEs are presented in Table 1. RTs are further depicted in Figure 3.

**Table 1:** Mean reaction times (RT in ms) and mean errors (PE in %) for Task 1 and Task 2 in Experiment 1.

		SOA	Near	Far
Task 1	RT	40	840 (30)	838 (33)
		90	828 (29)	836 (36)
		300	843 (36)	826 (37)
		900	876 (50)	875 (56)
	PE	40	4.8 (0.9)	4.5 (0.9)
		90	4.1 (0.7)	4.5 (0.9)
		300	3.6 (0.7)	3.5 (0.7)
		900	4.2 (0.7)	3.7 (0.8)
Task 2	RT	40	1152 (36)	1131 (32)
		90	1057 (34)	1055 (37)
		300	932 (37)	905 (34)
		900	680 (28)	665 (26)
	PE	40	5.3 (0.8)	6.1 (1.0)
		90	4.5 (0.8)	6.6 (1.1)
		300	5.5 (1.0)	5.6 (1.1)
		900	3.7 (0.7)	4.0 (0.9)


*Standard errors of the mean in parentheses. SOA, stimulus onset asynchrony.*

#### Task 1 RTs

There was no main effect of stimulus-hand proximity, *F* < 1. Also, we found no main effect of SOA, *F*(3,105) = 1.54, *p* = 0.188, $\eta_{p}^{2}$ = 0.05. Furthermore, we found no significant interaction of the factors stimulus-hand proximity and SOA, *F* < 1.

#### Task 1 PEs

There were no significant main effects of the factors stimulus-hand proximity, *F* < 1 and SOA, *F*(3,105) = 1.76, *p* = 0.160, $\eta_{p}^{2}$ = 0.05. There was further no significant interaction of stimulus-hand proximity and SOA, *F* < 1.

#### Task 2 RTs

We found no main effect of stimulus-hand proximity, *F* < 1. However, statistical analysis revealed a significant effect for the factor SOA, *F*(3,105) = 524.19, *p* < 0.001, $\eta_{p}^{2}$ = 0.94 revealing decreasing RTs with SOA increase. We observed no significant interaction of stimulus-hand proximity and SOA, *F* < 1.

#### Task 2 PEs

We observed no main effect of the factor stimulus-hand proximity, *F* < 1. A significant main effect of the factor SOA was found, *F*(3,105) = 5.14, *p* = 0.002, $\eta_{p}^{2}$ = 0.13, revealing decreased PEs with increasing SOA. No interaction of the factors stimulus-hand proximity and SOA was found, *F*(3,105) = 1.61, *p* = 0.192, $\eta_{p}^{2}$ = 0.04.

### Discussion

The results of Experiment 1 reveal two main findings. First, the characteristic dual task result pattern was identified, revealing the standard PRP effect: Task 1 RT and Task 1 PEs performance was not affected by temporal overlap between tasks, whereas Task 2 RTs and Task 2 PEs declined with increasing SOA (Pashler, 1994). Second, stimulus-hand proximity did not affect dual-task performance. No modulation of the PRP effect by stimulus-hand proximity was observed on the level of RTs and PE. Accordingly, the efficiency of the Task 1–Task 2 shifting process was not modulated in the near-hand space, at least not for a typical variant of the PRP dual-task paradigm. This indicates that the shifting operation in the PRP paradigm is quite robust against near-hand space-induced modulations of attention and cognitive control.

## Experiment 2

In Experiment 2 we performed a second dual-task experiment to investigate the effect of stimulus-hand proximity on Task 1–Task 2 switching using a PRP dual-tasking paradigm where Task 1–Task 2 switching had to occur between a color and a form-categorization task. A previous task-switching study found reduced switching costs when participants had to switch from a form-categorization task to a color-categorization task (Weidler and Abrams, 2014). In contrast, Experiment 1 involved a PRP dual-task paradigm in which shifts from Task 1 to Task 2 processing had to occur between two number-categorization tasks. It was our assumption, that distinct switching operations may be differentially susceptible to near-hand space. This assumption was further substantiated by a study in which subjects had to perform task-switches during a local/global task (i.e., judging either local or global aspects of objects) (Davoli et al., 2012). Contrary to the Weidler and Abrams (2014) experiment, the results provided by Davoli et al. (2012) revealed slower switching during near-hand, compared to far-hand, conditions. Thus, for Experiment 2, we adapted the switching operation of Experiment 1 by implementing a color categorization task for Task 2, while Task 1 remained unchanged (see Figure 1B). During Task 2, participants had to decide if the color of a rectangle was either orange or blue. The rest of the set-up and predictions were identical to Experiment 1.

### Methods

#### Participants

A sample of 36 participants from the University of Münster, Germany (24 female; *M*_age_ = 23.7 years, *SD* = 6.5) took part in the experiment. Participants received either course credits or monetary reward. All participants had normal or corrected-to-normal vision. All participants were right-handed. Written informed consent was provided by all participants prior to their inclusion in the experiment. One participant was excluded from further data analysis due to high error rates (*M*_total_ = 31.64%) surpassing 3 *SD*s of the overall total error rates (*M*_total_ = 9.26%). The remaining 35 subjects were included in for further data analysis.

#### Design

A 2 (Stimulus-hand proximity: near vs. far) × 4 (SOA: 40, 130, 300, and 900 ms) within-subjects repeated measures design was applied.

#### Stimuli, Apparatus, and Procedure

Task 1 in Experiment 2 was identical to Task 1 in Experiment 1. For Task 2, a blue and an orange colored rectangle served as stimuli (Stimulus 2). The position of stimulus presentation was the same as in Experiment 1. Subjects responded with the index (odd numbers) and middle finger (even numbers) of their right hand to Stimulus 1. The index (orange rectangle) and middle finger (blue rectangle) of their left hand was used to respond to Stimulus 2. The rest of the set-up was identical to Experiment 1, except that a chin rest was used to maintain a stable head position. The experiment had a near and far-hand condition, each comprising two blocks. One block contained 64 trials (16 trials per SOA). Thus, 128 trials were performed per stimulus-hand condition, which equals 256 trials in total. The rest of the procedure was identical to Experiment 1.

### Results

**FIGURE 4:**
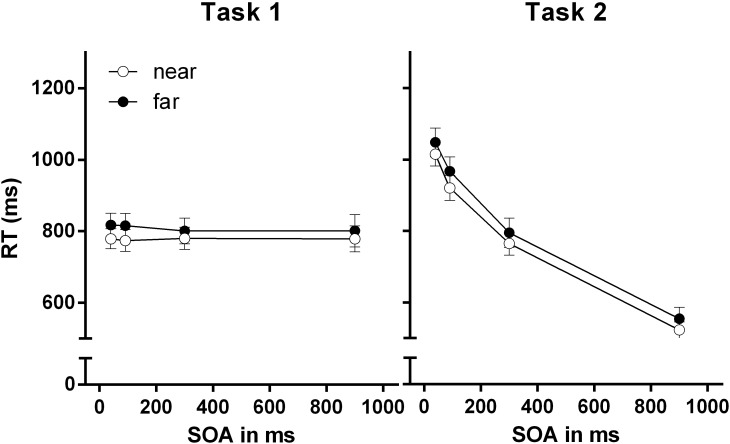
Reaction times for Task 1 and Task 2 for the near-hand and far-hand condition in Experiment 2. Error bars represent standard errors of the mean.

A repeated measures ANOVA was conducted on RTs and PEs in both tasks and included the factors stimulus-hand proximity, as well as SOA as within-subject factors. For RT analyses (Task 1 RTs and Task 2 RTs), error trials in either task (9.26%), and trials with RTs below 150 ms or above 3000 ms (<0.1%) were excluded prior to analysis. Double-errors (Task 1 and Task 2 errors) were excluded for Task 2 error analyses (<1.8%). Greenhouse–Geisser correction was applied in case of violation of sphericity. RTs and PEs are presented in Table 2. RTs are further depicted in Figure 4.

**Table 2:** Mean reaction times (RT in ms) and mean errors (PE in %) for Task 1 and Task 2 in Experiment 2.

		SOA	Near	Far
Task 1	RT	40	779 (28)	817 (33)
		90	774 (31)	816 (34)
		300	780 (31)	801 (36)
		900	779 (36)	801 (46)
	PE	40	4.3 (0.9)	5.7 (1.3)
		90	4.5 (1.0)	4.8 (1.0)
		300	3.1 (0.6)	3.3 (0.9)
		900	3.1 (0.6)	4.4 (1.0)
Task 2	RT	40	1016 (34)	1048 (40)
		90	921 (35)	967 (41)
		300	765 (32)	795 (41)
		900	523 (23)	555 (32)
	PE	40	6.0 (1.1)	5.2 (1.0)
		90	5.8 (1.2)	4.9 (0.8)
		300	5.2 (1.0)	4.7 (0.7)
		900	4.8 (1.1)	4.3 (0.7)


*Standard errors of the mean in parentheses. SOA, stimulus onset asynchrony.*

#### Task 1 RTs

The ANOVA revealed no significant effect of the main factor stimulus-hand proximity, *F*(1,34) = 1.44, *p* = 0.238, $\eta_{p}^{2}$ = 0.04. Also, no main effect was found for the factor SOA, *F* < 1. As well, there was no significant interaction of the factors stimulus-hand proximity and SOA, *F* < 1.

#### Task 1 PEs

There was no significant main effect of the factor stimulus-hand proximity, *F* < 1. The factor SOA was significant, *F*(3,102) = 3.84, *p* = 0.012, $\eta_{p}^{2}$ = 0.10, revealing decreasing PEs with SOA increase. No interaction of stimulus-hand proximity and SOA was found, *F* < 1.

#### Task 2 RTs

There was no main effect of stimulus-hand proximity, *F*(1,34) = 1.97, *p* = 0.170, $\eta_{p}^{2}$ = 0.06. A significant effect for the factor SOA was observed, *F*(3,102) = 493.31, *p* < 0.001, $\eta_{p}^{2}$ = 0.94, showing decreasing RTs with SOA increase. However, this effect was not affected by stimulus-hand proximity, *F* < 1.

#### Task 2 PEs

We found no significant main effect of the factor stimulus-hand proximity, *F* < 1. There was also no effect of the main factor SOA, *F* < 1. Furthermore, no significant interaction of SOA and stimulus-hand proximity was found, *F* < 1.

### Discussion

Using a PRP setup that required participants to switch to a color stimulus, we found Task 1 RTs to be unaffected by SOA, whereas Task 2 RTs showed an increase of RTs with decreasing SOA – a typical PRP effect. For PEs, we found a slight increase of error rates at short SOA, which was significant for Task 1 PEs and suggests increased difficulty of dual-task processing at high temporal task overlap. Importantly, this finding was not affected by stimulus-hand proximity. We did not find a modulation of the PRP effect by hand position. Simply put, Task 1–Task 2 switching was not altered in near-hand space. Therefore, our results suggest a robustness of the PRP shifting operation toward modulations of attention and cognitive control induced by the near-hand space. This finding is different to previous work showing that the shift between two different tasks in the task-switching paradigm is optimized in near-hand space (Weidler and Abrams, 2014).

## Experiment 3

In Experiment 3 we performed a third dual-task experiment to investigate the effect of stimulus-hand proximity on Task 1–Task 2 switching using a task setup where Task 1 and Task 2 referred to different features of a single stimulus. It is conceivable that the previous absence of a PRP modulation by stimulus-hand proximity may be due to a frequent feature of dual tasking – the presence of two stimuli that have to be concurrently processed. Instead, in task-switching studies participants often have to switch between different features of a single stimulus. This assumption is supported by the fact that two judgments concerning two features of one single object are facilitated compared to two judgments concerning two distinct objects (Duncan, 1984). The latter suggests that distinct switching operations have to be performed when processing two dimensions of one single object in close temporal succession compared to processing two distinct objects. Thus, the reduced switch-costs observed in the Weidler and Abrams (2014) experiment may be traced back to the particular feature of using one single bivalent stimulus (i.e., colored geometrical figures) on which two different judgments had to be performed in alternation. In order to adapt the task setup to the study of Weidler and Abrams (2014) while keeping the core logic of a PRP dual task, we used a single bivalent stimulus for Task 1 and Task 2 whereby Task 1–Task 2 switching referred to different features of one single stimulus. To do this, the number stimulus relevant for Task 1 changed its color requiring a color categorization for Task 2, see Figure 1C. All predictions were the same as in our previous experiments.

### Methods

#### Participants

A new sample of 35 participants from the University of Münster, Germany (27 female; *M*_age_ = 24.4 years, *SD* = 4.3) took part in the experiment. Participants received either course credits or monetary reward. All participants had normal, or corrected-to-normal, vision. All participants were right handed. Written informed consent was provided by all participants prior to their inclusion in the experiment. One participant was excluded from further data analysis due to error rates (*M*_total_ = 32.81%) exceeding 3 *SD*s of the overall total error rates (*M*_total_ = 9.56%). The remaining 34 participants were included for further data analysis.

#### Design

A 2 (Stimulus-hand proximity: near vs. far) × 4 (SOA: 40, 130, 300, and 900 ms) within-subjects repeated measures design was applied.

#### Stimuli and Apparatus, and Procedure

In Experiment 3, the two tasks used in Experiment 2 were presented centrally on the screen as a single bivalent stimulus. Thereby, Task 1 was identical to Experiments 1 and 2 (number categorization). Task 2 was the same color categorization task as in Experiment 2 (orange vs. blue). Stimulus 1 was presented centrally, and subsequently changed its color thereby initiating Stimulus 2 presentation. The rest of the set-up was identical to Experiment 2. The experiment had a near and far-hand condition, each comprising two blocks. One block contained 64 trials (16 trials per SOA). Thus, 128 trials were performed per stimulus-hand condition, which equals 256 trials in total. The rest of the procedure was identical to Experiment 2.

### Results

**FIGURE 5:**
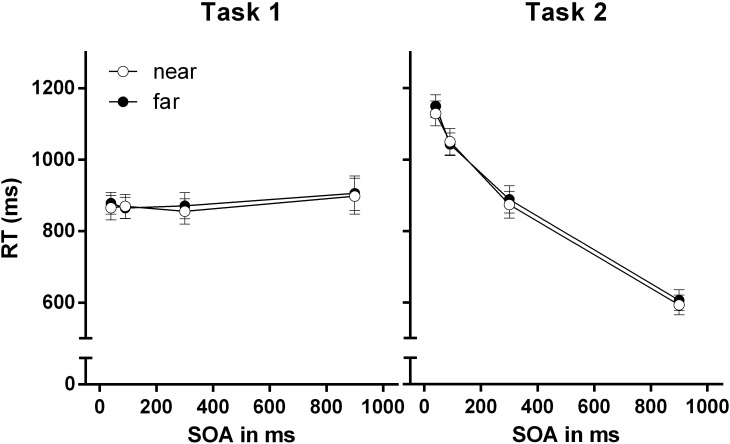
Reaction times for Task 1 and Task 2 for the near-hand and far-hand condition in Experiment 3. Error bars represent standard errors of the mean.

A repeated measures ANOVA was conducted on RTs and PEs in both tasks. Like in Experiments 1 and 2, within-subject factors were stimulus-hand proximity and SOA. Error trials in either task (9.56%), and trials below 150 ms and above 3000 ms (<0.1%) were excluded from RT data analysis. Double-errors (Task 1 and Task 2 errors) were excluded for Task 2 error analyses (<2%). Greenhouse–Geisser correction was applied in case of violation of sphericity. RTs and PEs are presented in Table 3. RTs are further depicted in Figure 5.

**Table 3:** Mean reaction times (RT in ms) and mean errors (PE in %) for Task 1 and Task 2 in Experiment 3.

		SOA	Near	Far
Task 1	RT	40	865 (34)	878 (31)
		90	869 (34)	865 (30)
		300	855 (35)	871 (36)
		900	898 (50)	906 (48)
	PE	40	6.0 (1.0)	6.3 (1.0)
		90	6.9 (1.1)	5.4 (1.1)
		300	4.1 (0.8)	4.2 (1.0)
		900	4.1 (1.1)	3.6 (1.0)
Task 2	RT	40	1129 (35)	1150 (32)
		90	1050 (37)	1043 (32)
		300	874 (37)	889 (38)
		900	593 (28)	606 (29)
	PE	40	3.8 (0.7)	5.4 (0.9)
		90	4.5 (0.8)	3.9 (0.8)
		300	4.0 (0.7)	4.1 (0.6)
		900	5.2 (0.7)	4.9 (0.9)


*Standard errors of the mean in parentheses. SOA, stimulus onset asynchrony.*

#### Task 1 RTs

Neither a main effect for stimulus-hand proximity, *F* < 1, nor for SOA, *F*(3,99) = 1.48, *p* = 0.236, $\eta_{p}^{2}$ = 0.04, was found. Moreover, no interaction of both factors was found, *F* < 1.

#### Task 1 PEs

No main effect of stimulus-hand proximity was found, *F* < 1. There was a main effect of SOA, *F*(3,99) = 5.61, *p* = 0.003, $\eta_{p}^{2}$ = 0.15, revealing decreasing PEs with SOA increase. There was no interaction of both factors, *F* < 1.

#### Task 2 RTs

We found no effect of stimulus-hand proximity, *F* < 1. However, we found a main effect for SOA, *F*(3,99) = 425.25, *p* < 0.001, $\eta_{p}^{2}$ = 0.93, revealing decreasing RTs with SOA increase. We found no interaction of stimulus-hand proximity and SOA, *F* < 1.

#### Task 2 PEs

Analysis revealed no main effect of stimulus-hand proximity and SOA, *Fs* < 1. As well, we found no interaction of stimulus-hand proximity and SOA, *F*(3,99) = 1.31, *p* = 0.276, $\eta_{p}^{2}$ = 0.04.

### Discussion

In Experiment 3 we found evidence for the classic PRP effect when both tasks referred to different features of a single stimulus. Task 2 RTs increased with decreasing SOA between the number presentation and its color change. As expected, Task 1 RTs were unaffected by SOA. Higher Task 1 PEs were found at short SOA indicating increased dual-task difficulty at high task overlap. In Experiment 3, we found no modulation of the PRP effect by stimulus-hand proximity when using a single bivalent stimulus. The Task 1–Task 2 shifting operation was not altered in the near-hand space in an adaptation of the typical PRP paradigm with bivalent stimuli where a shift from number to color information was required. Most importantly, the findings illustrate that Task 1–Task 2 shifting in dual-tasking is unaffected by the near-hand space. Thus, our findings suggest that the shifting process during the PRP paradigm is a relatively rigid processes, which cannot be manipulated via acute changes in attention and cognitive control when two stimuli are processed in near-hand space. The results of Experiment 3 show that the shifting mechanisms involved in the PRP paradigm and the task-switching paradigm have different sensitivities to hand nearness manipulations. This may either be due to different cognitive switching operations required in both paradigms or may be traced back to other more methodological and paradigm-specific differences.

## General Discussion

The aim of this study was to investigate the effect of the near-hand space on between-task switches in three different PRP paradigms. The main finding from the series of three experiments is that the size of the PRP effect did not change with near, compared to far, stimulus-hand proximity. This indicates that the near-hand space did not alter Task 1–Task 2 shifting in the PRP dual-task paradigm. The classic PRP effect in each of the three experiments was shown by deteriorating Task 2 performance at shorter SOA while Task 1 was relatively unaffected by task-overlap.

In Experiment 1, we tested a PRP paradigm that was adapted from the PRP literature (Fischer et al., 2007) in near and far-hand conditions. Specifically, Task 1 was a number categorization task (odd vs. even) and Task 2 was a letter categorization task (vowel vs. consonant). Stimulus 1 and Stimulus 2 presentation was spatially segregated, with Stimulus 1 presented on the right side of the screen and Stimulus 2 presented on the left side of the screen. Based on the available evidence, we hypothesized that near-hand conditions would lead to either prolonged Task 1–Task 2 switching due to delayed attentional disengagement (Abrams et al., 2008) or to improved Task 1–Task 2 switching due to an increased level of cognitive control eliciting an attentional benefit of processing both stimuli (Weidler and Abrams, 2014). The finding that stimulus-hand proximity did not affect Task 1–Task 2 switching was not in line with our predictions and suggests that the switching operation is not altered in the near-hand space in classical dual-tasks. In Experiment 2, we tested the effect of stimulus-hand proximity on Task 1–Task 2 switching, this time using an adapted PRP paradigm where Task 2 was replaced by a color-categorization task, while Task 1 number categorization remained identical to Experiment 1. The finding that Task 1–Task 2 switching was unaffected by stimulus-hand proximity is surprising as previous work has provided evidence for reduced switch costs in a task-switching paradigm that required participants to switch between a form and a color task (Weidler and Abrams, 2014). Our findings may indicate that the switching operation between two distinct and spatially segregated stimuli is not sensitive to near-hand effects. Therefore, in Experiment 3, we integrated Stimulus 1 and Stimulus 2 into a single centrally presented bivalent stimulus manipulating stimulus-hand proximity. Again, we did not observe any effect of stimulus-hand proximity on Task 1–Task 2 switching. Task 1–Task 2 switching was not affected by hand proximity, even when the switching operation had to be performed between two different aspects of a single stimulus rather than between two tasks each referring to a separate stimulus. Thus, again, we were not able to identify an effect of the near-hand space on PRP dual-task performance.

Together, the three experiments reported in this study seem to indicate that between-task switching operations are not affected by the near-hand space. This was apparent during two classic PRP set-ups, in which two distinct stimuli were presented, and during a task set-up in which a single bivalent stimulus had to be processed. Overall, according to our three experiments, it seems very unlikely that the near-hand space does alter Task 1–Task 2 shifting in the PRP paradigm. This is surprising, as previous studies have revealed either delayed attentional disengagement (Abrams et al., 2008) or improved cognitive control (Weidler and Abrams, 2014) in task set-ups that required the switching between two consecutive stimuli (Abrams et al., 2008) or tasks (Weidler and Abrams, 2014). Consequently, shifting operations in the PRP paradigm seem to be relatively rigid and resistant to acute cognitive modulations typically induced by a hand proximity manipulation (Tseng et al., 2012; Brockmole et al., 2013; Abrams et al., 2015). The reduced switch costs reported by Weidler and Abrams (2014) are generally unlikely to reflect altered task preparation costs, as the preparation phase (cue-stimulus interval) was constantly set to 1000 ms in the study of Weidler and Abrams (2014). Rather, the reduced costs may reflect reduced residual switch costs (Monsell, 2003), which have previously been proposed to reflect a structural limitation (Vandierendonck et al., 2010), and have also been proposed for the PRP paradigm (Pashler, 1994). This may also support the assumption that shifting operations in the PRP paradigm are a relatively rigid process in general (Pashler, 1994). The more astonishing it seems that we did not find a reduced PRP effect under hands proximal conditions, which would mimic the findings of reduced switch costs under proximal conditions (Weidler and Abrams, 2014). This suggests that the attentional benefit of processing stimuli in near hand space does not extend to an entire Stimulus 1- Stimulus 2 compound in the PRP paradigm, in which both stimuli appearing in the space between both hands are optimized since we did not find an optimized shifting between both stimuli and tasks.

Another question that arises is, what do these findings tell us about the commonalities and differences between the shifting operations in various dual-task paradigms more generally? The shift in the attentional blink task refers to a switch between two separate stimuli that are presented within a short temporal interval and the stimuli are presented at a single location. As we found no delayed disengagement from processing Stimulus 1 in the near-hand space in none of our experiments, our results clearly contrast the results of Abrams et al. (2008), where the near-hand space induced a slower disengagement from processing Stimulus 1. While switching in the attentional blink task is related to an attention switching between different stimuli, the switching operation in the PRP involves the preparation and switching to an entire new task set involving a much more complex switching operation also including the activation of the new response set. This finding is of particular interest as it has been suggested that similar neural mechanisms may underlie both PRP processing and the attentional blink effect (Marois and Ivanoff, 2005; Marti et al., 2012).

A critical difference between more classical dual tasks (like the PRP paradigm) and task switching is that many task-switching studies use cue-based task switching. The study of Weidler and Abrams (2014) used a form of cue-based switching in which each trial began with a rectangular outline (solid or dashed) that indicated which task had to be performed (i.e., color or shape categorization). The absence of an effect of the near-hand space on PRP dual-task performance in our three experiments may suggest that it might not be the task-switching operation itself that is improved under near-hand conditions. Instead, one could speculate that the observed reduction of task-switching costs under near-hand conditions may be traced back to changes in the processing of the task cue that indirectly affects the size of task switching costs. Near-hand conditions, for example, may have induced a more in-depth cue processing, enhancing the preparation process and leading to reduced task switching costs.

## Conclusion

Our findings contribute novel and important knowledge to the domain of the near-hand space in multitasking research. We found between-task switches to be unaltered by the near-hand space, indicating that this form of dual-task performance is not altered when hands are located near the stimuli. Mechanistically, it appears that neither a delayed disengagement of attention, nor an enhancement of cognitive control seemed to alter PRP performance. Also, our results suggest that, despite obvious mechanistic similarities between diverse dual-tasking paradigms (Koch et al., 2018), there may at least be some processes that differ substantially between the attentional blink, the task switching paradigm and the PRP paradigm. From thus we conclude that, while a large number of attentional and cognitive effects seem to be sensitive to manipulations of embodied cognition such as our hand proximity manipulation (Reed et al., 2006; Abrams et al., 2008; Gozli et al., 2012; Weidler and Abrams, 2014; Liepelt and Fischer, 2016), we think that it is important to show that some cognitive operations seem to be quite robust and relatively independent of hand proximity. Our findings are not only relevant for basic research on multitasking and the near-hand space, but also for more applied dual-task settings. Our findings suggest that an optimization of task switching in handheld devices may not be easily achieved. However, our findings also indicate that the switching operation between tasks is not disturbed when processing multiple stimuli in the vicinity of our hands in modern handheld devices. Future research should test for effects of hand proximity in multitasking scenarios with various task demands and further levels of attentional control besides those involved in handling cognitive capacity limitations (Pashler, 1994; Meyer and Kieras, 1997). As multitasking involves a diverse set of cognitive control operations representing a multifaceted phenomenon, our results do not exclude alterations of other functions involved in multitasking performance through manipulations of stimulus-hand proximity.

## Author Contributions

TH wrote the manuscript with the support of RL, RF, and JP. RL and RF designed the experiments. JP carried out the experiments. TH performed the statistical analysis.

## Conflict of Interest Statement

The authors declare that the research was conducted in the absence of any commercial or financial relationships that could be construed as a potential conflict of interest.
